# “Back to Braak”: Role of Nucleus Reuniens and Subcortical Pathways in Alzheimer’s Disease Progression

**DOI:** 10.14283/jpad.2024.42

**Published:** 2024-02-21

**Authors:** S. Censi, C. Sestieri, M. Punzi, A. Delli Pizzi, A. Ferretti, F. Gambi, V. Tomassini, Stefano Delli Pizzi, Stefano L. Sensi, Alzheimer’s Disease Neuroimaging Initiative

**Affiliations:** 1 Department of Neuroscience, Imaging, and Clinical Sciences, University «G. d’Annunzio» of Chieti-Pescara, Via Polacchi, 11, Chieti, 66100 Italy; 2 Institute for Advanced Biomedical Technologies (ITAB), “G. d’Annunzio” University, Chieti-Pescara, Italy; 3 Department of Innovative Technologies in Medicine and Dentistry, «G. d’Annunzio» University of Chieti-Pescara, Chieti, Italy; 4 UdA-TechLab, Research Center, University “G. d’Annunzio” of Chieti-Pescara, 66100 Chieti, Italy; 5 MS Centre, Institute of Neurology, SS Annunziata University Hospital, Chieti, Italy; 6 Molecular Neurology Unit, Center for Advanced Studies and Technology (CAST), University «G. d’Annunzio» of Chieti-Pescara, Chieti-Pescara, Italy

**Keywords:** Alzheimer’s Disease (AD), Mild Cognitive Impairment (MCI), thalamus, reuniens, Magnetic Resonance Imaging (MRI)

## Abstract

**Background:**

Patients with Alzheimer’s Disease (AD) exhibit structural alterations of the thalamus that correlate with clinical symptoms. However, given the anatomical complexity of this brain structure, it is still unclear whether atrophy affects specific thalamic nuclei and modulates the clinical progression from a prodromal stage, known as Mild Cognitive Impairment (MCI), to full-fledged AD.

**Objectives:**

To characterize the structural integrity of distinct thalamic nuclei across the AD spectrum, testing whether MCI patients who convert to AD (c-MCI) show a distinctive pattern of thalamic structural alterations compared to patients who remain stable (s-MCI).

**Design:**

Investigating between-group differences in the volumetric features of distinct thalamic nuclei across the AD spectrum.

**Setting:**

Prodromal and clinical stages of AD.

**Participants:**

We analyzed data from 84 healthy control subjects (HC), 58 individuals with MCI, and 102 AD patients. The dataset was obtained from the AD Neuroimaging Initiative (ADNI-3) database. The MCI group was further divided into two subgroups depending on whether patients remained stable (s-MCI, n=22) or progressed to AD (s-MCI, n=36) in the 48 months following the diagnosis.

**Measurements:**

A multivariate analysis of variance (MANOVA) assessed group differences in the volumetric features of distinct thalamic nuclei obtained from magnetic resonance (MR) images. A stepwise discriminant function analysis identified which feature most effectively predicted the conversion to AD. The corresponding predictive performance was evaluated through a Receiver Operating Characteristic approach.

**Results:**

AD and c-MCI patients showed generalized atrophy of thalamic nuclei compared to HC. In contrast, no significant structural differences were observed between s-MCI and HC subjects. Compared to s-MCI, c-MCI individuals displayed significant atrophy of the nucleus reuniens and a trend toward significant atrophy in the anteroventral and laterodorsal nuclei. The discriminant function analysis confirmed the nucleus reuniens as a significant predictor of AD conversion, with a sensitivity of 0.73 and a specificity of 0.69.

**Conclusions:**

In line with the pathophysiological relevance of the nucleus reuniens proposed by seminal post-mortem studies on patients with AD, we confirm the pivotal role of this nucleus as a critical hub in the clinical progression to AD. We also propose a theoretical model to explain the evolving dysfunction of subcortical brain networks in the disease process.

**Electronic Supplementary Material:**

Supplementary material is available for this article at 10.14283/jpad.2024.42 and is accessible for authorized users.

## Introduction

**A**lzheimer’s disease (AD) is one of the most common age-related neurodegenerative diseases, with a growing incidence worldwide. The clinical manifestations usually initiate with a prodromal state known as Mild Cognitive Impairment (MCI), which may eventually progress to subsequent AD in a fraction of subjects (10–15%). Since the neurodegenerative process leading to AD begins more than a decade before a clinical diagnosis can be made (1), the detection of early signs of the future conversion from MCI to AD is of great clinical importance for the decision to initiate both pharmacological and non-pharmacological interventions. Magnetic Resonance Imaging (MRI) is a non-invasive tool for exploring macrostructural changes across the AD spectrum (2–4). In particular, atrophy and metabolic alterations in the hippocampal and entorhinal regions have emerged as potential clinical markers of the progression to AD (5–7). This emphasis is coherent with the pivotal role of the hippocampus in episodic memory and navigation (8), two cognitive domains that are significantly impacted in early AD. However, MRI research also demonstrates the involvement of other cortical and subcortical networks, like the Papez circuit, in the neurodegenerative process leading to AD (9).

Growing lines of evidence indicate that patients with AD also exhibit significant structural alterations of the thalamus (10–13), pathological features that correlates with clinical symptoms (9, 14). However, previous studies treated the thalamus as a whole, potentially neglecting its constituent nuclei’s anatomical and functional specificity. Anatomically, the thalamus is a heterogeneous structure comprising several nuclei, each bidirectionally connected to distinct subcortical and cortical regions (15). Post-mortem AD studies revealed a hierarchical pathological evolution within thalamic nuclei, indicating abnormal accumulation of tau protein, especially in nuclei associated with the limbic system, such as the anterior and lateral nuclei (16). While the AD model based on amyloid deposition is highly debated (17, 18), tau-driven pathological processes are considered more fit to indicate the regional progression of the disease and its impact on symptoms. In addition, the classical model of tau pathology spreading in AD, developed by Braak and Braak (19, 20), has highlighted the possible pathophysiological relevance of the nucleus reuniens. This structure exhibits an interesting pattern of anatomical connections with cortical and subcortical regions typically affected by AD, including the hippocampal formation and the medial prefrontal cortex (21).

Despite these seminal post-mortem observations, the spatial pattern of structural integrity of the thalamus across the AD spectrum remains largely unexplored. One reason for this knowledge gap is that the structure’s composition complicates the structural analysis of the thalamus via MRI, as the individual nuclei cannot be easily separated using standard analysis methods of anatomical images. To overcome these limitations, we combined probabilistic techniques and a priori information derived from ex-vivo MRI and histology to analyze MR raw images obtained from the AD Neuroimaging Initiative (ADNI) platform and perform an accurate parcellation of thalamic nuclei (22). This innovative approach has demonstrated test-retest solid reliability and robustness when applied to data involving elderly healthy subjects and/or AD patients (23). We characterized the structural integrity of distinct thalamic nuclei across the AD spectrum and tested whether MCI patients who convert to AD (c-MCI) exhibit a distinctive pattern of thalamic structural alterations compared to patients who remain stable (s-MCI).

## Material and Methods

### Study Data, Inclusion, and Diagnostic Criteria

All data for this article were obtained from the ADNI-3 database, an ongoing large-scale, multi-center study across the U.S. and Canada (adni.loni.usc.edu). ADNI-3 was launched in late 2016 as a public/private partnership to identify brain imaging [MRI, Positron Emission Tomography (PET)] and clinical, cognitive, and molecular biomarkers of AD and aging. For updated information on the initiative, see www.adniinfo.org. The study was performed according to ethical standards and the Declaration of Helsinki (1997). Informed consent was obtained from study participants or legally authorized representatives. Details on the study protocol are reported on the ADNI website (http://www.adni-info.org).

A total of 244 subjects were included in this study, divided into 84 HC, 58 patients with MCI, and 102 patients with probable AD. The HC group included cognitively normal individuals from baseline to follow-up 48 months. MCI subjects were further divided based on clinical follow-up into 35 subjects who converted to AD within 48 months (c-MCI) and 22 subjects who did not convert to AD within 48 months (s-MCI). The Detailed demographic characteristics are summarized in Table 1. The age range was between 55 and 95 years.

**Table 1:** Demographic features and neuropsychological features of study groups at baseline

**Variable**	**HC**		**s-MCI**		**c-MCI**		**AD**		**Group comparison**		
									*X*^2^	**p**	
**N (% male)**	**84 (47%)**		**22 (50%)**		**36 (57%)**		**102 (58%)**		**1.058**	**0.304**	
	**Mean**	**SD**	**Mean**	**SD**	**Mean**	**SD**	**Mean**	**SD**	**F**	**p**	**s-MCI vs. c-MCI**
Age	75.0	6.8	76.2	6.2	75.1	8.2	76.6	8.7	0.368	0.776	NA
Scholarity (y)	17.3	2.3	16.9	3.2	16.2	2.3	15.5	2.5	2.061	0.112	NA
CDR-SB	0.0	0.0	1.3	1.2	2.1	1.1	5.1	2.4	94.93	**PP**	.066
ADAS-11	5.0	2.7	8.6	4.2	12.2	4.3	20.5	7.3	41.40	**PP**	**.025**
ADAS-13	7.6	4.1	13.1	6.2	20.3	6.1	31.2	8.9	64.59	**PP**	**PP**
ADAS-Q4	2.3	1.7	3.8	2.3	7.0	2.3	8.8	1.5	85.23	**PP**	**PP**
MMSE	29.3	0.8	28.7	1.8	26.6	2.3	22.1	4.1	63.18	**PP**	**.004**
MoCA	26.9	2.4	23.5	4.0	21.6	3.3	16.6	4.9	35.52	**PP**	.135
FAQ	0.2	0.7	2.4	3.0	6.5	4.4	15.3	7.6	65.36	**PP**	**.009**
RAVLT-IR	48.3	11.1	39.4	11.9	28.7	9.2	22.9	7.1	38.86	**PP**	**.001**
RAVLT-L	5.6	2.5	5.2	3.1	3.0	2.0	1.9	1.8	12.03	**PP**	**.007**
RAVLT-DR	8.9	4.2	5.7	4.5	1.7	2.3	0.5	1.5	50.51	**PP**	**PP**
RAVLT-TOT	13.7	1.9	12.3	2.9	9.1	3.9	6.2	4.3	30.51	**PP**	**.005**
RAVLT-RN	12.9	2.4	11.0	3.3	6.5	4.5	3.6	3.7	50.92	**PP**	**PP**
LM-IR	15.5	3.2	12.5	4.9	7.8	4.0	4.7	3.4	47.89	**PP**	**PP**
LM-DR	14.6	3.6	11.0	4.8	4.9	4.1	1.8	2.7	79.29	**PP**	**PP**
CT-Drawing	4.8	0.4	4.2	1.0	4.3	0.8	3.4	1.5	6.74	**PP**	.991
CT-Copy	4.8	0.5	4.6	0.6	4.7	0.6	3.9	1.5	1.55	0.208	NA
TMT-A	29.3	8.5	33.0	15.0	44.8	21.8	64.4	38.4	6.67	**PP**	.244
TMT-B	65.5	29.3	93.2	54.0	127.7	50.8	189.5	94.2	19.87	**PP**	.432
AF	22.5	4.9	18.6	5.2	15.7	4.4	12.1	5.1	21.31	**PP**	.139
MINT-CUE	0.3	0.7	0.4	0.9	0.7	1.4	1.0	3.2	2.28	0.086	NA
MINT-Total	30.6	3.7	27.9	7.1	28.0	4.2	24.5	7.8	4.51	**0.006**	.994
MINT-UNC	30.7	1.9	28.9	3.9	27.2	4.6	24.8	6.2	7.41	**PP**	.748
NPI-total	1.2	2.8	2.5	3.4	4.4	5.8	10.4	11.1	7.74	**PP**	.804
GDS	0.8	1.1	2.0	1.9	1.8	1.7	2.2	1.9	8.01	**PP**	.990

Values are expressed as the mean &plusmn; standard deviation (SD). Bold values are statistically significant comparisons. Abbreviations: AD=Alzheimer’s disease; ADAS=Alzheimer’s Disease Assessment Scale (11 items and 13 items versions); ADAS-Q4=ADAS delayed word recall subscale; AF=Animal Fluency; RAVLT-TOT=Rey’s Auditory Verbal Learning Total Recognition score; CDR-RS=Clinical Dementia Rating Scale; CT-Copy=Clock Test- Copy score; CT-Drawing=Clock Test- Drawing total score; c-MCI=patients with MCI who convert to AD within 48-month follow-up; FAQ=Functional Activities Questionnaire; GDS=Geriatric Depression Scale; HC=healthy control stable after 48 months of follow-up; LM-IR=Logical Memory-Immediate Recall Total Number of Story Units Recalled; LM-DR=Logical Memory-Delayed Recall Total Number of Story Units Recalled; MINT-CUE=Multilingual Naming Test Total Correct - with Semantic Cue; MINT-Total=Multilingual Naming Test Total Correct (Uncued + Correct with Semantic cue); MINT-UNC =Multilingual Naming Test Total Uncued Correct; MMSE=Mini-Mental State Examination Total Score; MoCA=Montreal Cognitive Assessment; NA=Not Applicable; NPI=Neuropsychiatric Inventory Questionnaire; RAVLT-IR=Rey’s Auditory Verbal Learning Test, Immediate Recall (sum of 5 trials); RAVLT-L=Rey’s Auditory Verbal Learning Test, learning (trial 5 - trial 1); RAVLT-DR=Rey’s Auditory Verbal Learning Test, 30 minute Delayed Recall; RAVLT-RN=Rey’s Auditory Verbal Learning Test Delayed Recognition (RAVLT TOT Recognition Score - Total Intrusions); RAVLT-TOT= Rey’s Auditory Verbal Learning Test Total Recognition Score; s-MCI=MCI patients who did not convert to AD after 48-month follow-up; TMT=Trail Making Test (parts A and B); t-Tau=CSF Total Tau concentration (pg/mL).

T1-weighted images were acquired by a 3T scanner using a harmonized protocol and identical acquisition parameters to minimize site differences (https://adni.loni.usc.edu/methods/mri-tool/mri-analysis/). In our study, we included only those subjects who completed baseline 3D T1-weighted scans and neuropsychological/clinical investigations. Subjects who underwent MRI but had incomplete clinical and demographic information and those whose MRI scan had technical issues (severe motion, missing volumes, or corrupted files) were excluded from the study sample.

### Clinical assessments

At the time of the MRI scan, all participants were extensively assessed for cognitive functions with the Mini Mental State Examination (MMSE) (24) and the Montreal Cognitive Assessment (MoCA) (25) to evaluate global cognition; Functional Activities Questionnaire (FAQ) for the investigation of daily living activities (26); the Cognitive subscales of the Alzheimer’s Disease Assessment Scale (ADAS-11 items scores; ADAS-13 items scores; ADAS-Q4 delayed word recall subscale) to study the severity of impairments of memory, learning, language, praxis, and orientation (27, 28); the Clock Drawing Test (CT-Drawing) to assess dysfunction of visuoconstructive abilities (29); the Animal Fluency [30] and Multilingual Naming Test (MINT) to detect language capacities and naming deficits (31); the Trail Making Test (time to completion, TMT parts A and B) for processing speed and executive function (32); the Rey Auditory Verbal Learning Test (RAVLT) to investigate auditory verbal learning and memory (immediate memory span, learning, delayed recall, and delayed recognition) and the Logical Memory II subscale (LM) of the Wechsler Memory Scales Revised (WMS-R) Story A to assess immediate and delayed recall (33, 34); the Neuropsychiatric Inventory (NPI) and the Geriatric Depression Scale (GDS) to obtain information on psychopathological and behavioral features (35, 36).

The HC subjects exhibited no significant impairment in cognitive functions or activities of daily living. Their MMSE scores fell within the range of 27 to 30. They achieved a global score of 0 on the Clinical Dementia Rating Scale (CDR-RS) (37), with a specific score of 0 in the Memory Box category, indicating a lack of dementia. Moreover, their memory function was assessed using the LM, and they scored above the education-adjusted cutoffs (9 for 16 or more years of education, 5 for 8–15 years of education, 3 for 0–7 years of schooling), confirming normal memory performance.

For subjects with MCI, the inclusion criteria involved MMSE scores between 24 and 30, subjective memory concerns, and memory impairments identified by their partners. Moreover, the CDR Memory Box score for MCI subjects was required to be at least 0.5, indicating a mild cognitive decline. Memory function was evaluated using the LM, and MCI subjects scored below education-adjusted cut-offs (< 11 for 16 or more years of education; ≤ 9 for 8–15 years of schooling; ≤ 6 for 0–7 years of education), confirming abnormal memory performance. However, their general cognitive status and functional abilities were preserved enough to exclude a diagnosis of AD.

Subjects of the AD group met the criteria for probable AD as set by the National Institute of Neurologic and Communicative Disorders and Stroke, as well as the Alzheimer’s Disease and Related Disorders Association.

For more details about the ADNI-3 inclusion criteria see https://adni.loni.usc.edu/wp-content/themes/freshnews-dev-v2/documents/clinical/ADNI-3_Protocol.pdf.

The clinical determination of the conversion from MC to dementia was made by experienced ADNI clinicians. This determination relied on a comprehensive evaluation, including information from the patient and a well-informed caregiver. Additionally, biological markers and neuropsychological assessments were considered to support the diagnosis.

### MRI data acquisition and analysis

**Figure 1:**
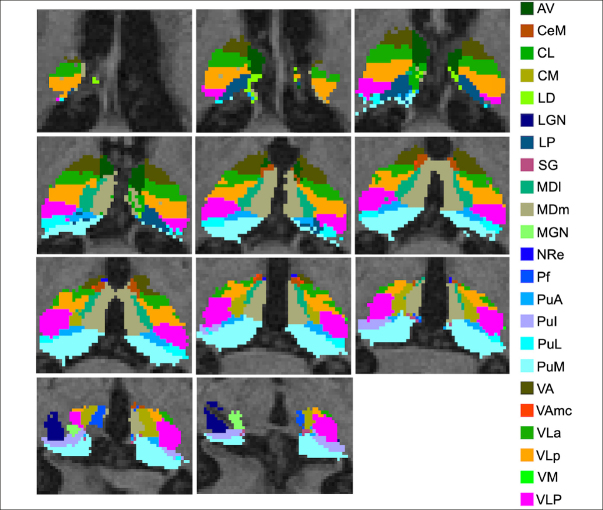
Representative thalamic nuclei parcellation of a study participant

The imaging protocol included a 3T T1-weighted sagittal 3D MPRAGE volume (voxel size 1.05×1.05×1.2 mm). A detailed ADNI data acquisition protocol description can be found at https://adni.loni.usc.edu/methods/documents/mriprotocols/. T1-weighted images were processed with FSL-FIRST’s pipeline (https://fsl.fmrib.ox.ac.uk/fsl/fslwiki/FIRST) to obtain an accurate volumetry of the entire thalamus (38). T1-weighted images were processed with FreeSurfer 7.3 using the «recon-all -all» command line. Briefly, this processing includes motion correction and intensity normalization of T1-weighted images, removal of non-brain tissue using a hybrid watershed/surface deformation procedure, automated Talairach transformation, segmentation of the subcortical white matter and deep gray matter volumetric structures (including the hippocampus, amygdala, caudate, putamen, ventricles), tessellation of the gray matter white matter boundary, and derivation of cortical thickness. The tool provided an automated reconstruction and labeling of cortical and subcortical regions and a measure of estimated Intracranial Volume (eTIV). Based on the methods developed by Iglesias and colleagues (2018), the thalamus of each subject was parceled in twenty-five nuclei for each hemisphere (Figure 1). Specifically, the anterior group included the anterior (AV), the laterodorsal (LD), and lateroposterior (LP) nuclei; the medial groups consisted of the paratenial (Pt), medial ventral reuniens (MV-re), magnocellular medial mediodorsal (MDm) and parvocellular lateral mediodorsal (MDl) nuclei; the pulvinar regions included the anterior (PuA), inferior (PuI), lateral (PuL), medial (PuM) nuclei; the metathalamus encompassed the medial (MGN) and lateral (LGN) geniculate nucleus, the nucleus limitans (SG); the ventral portion consisted of the ventral-anterior (VA) nucleus, ventrolateral anterior (VLa) and posterior (VLp) regions, and the ventral-postero-lateral and ventromedial nuclei; the non-specific nuclei included the central medial (CeM), central lateral (CL), paracentral (Pc), centromedian (CM), and parafascicular (Pf) nuclei. We thoroughly examined all the segmentations of thalamic nuclei and found no issues. It is noteworthy that no additional manual corrections were made, as the anatomical boundaries consistently matched the atlas and remained uniform across subjects, a result confirmed by meticulous visual inspection.

The images are presented in the neuroradiological convention.

### Statistical analysis

Analysis of variance and Turkey’s post hoc tests were used to evaluate the group differences regarding demographic, neuropsychological, and clinical data. Categorical variables were analyzed using chi-square tests. For the MRI measures, a multivariate analysis of variance (MANOVA) with 4 levels [HC, s-MCI, c-MCI, AD], followed by Turkey’s post-hoc comparison, was applied to test the differences among groups. The MRI volumes of the thalamic nuclei and the whole thalamus were additionally included in a stepwise discriminant function analysis to determine whether a set of variables effectively predicted category membership (s-MCI or c-MCI). Wilk’s lambda tests how well each independent variable level contributes to the model. Each independent variable is tested by putting it into the model and then taking it out — generating a Wilks’ lambda statistic. The significance of changes in Wilks’ lambda is measured with an F-test; if the F-value is greater than the critical value, the variable is kept in the model. Finally, a Receiver Operating Characteristic (ROC) analysis of the predictive function was used to determine a relative sensitivity and specificity cut-off to investigate whether the baseline volumes of specific subfields could discriminate MCI subjects that progress to AD. ROC analysis allows for the comprehensive evaluation of classification performance by assessing the trade-off between true positive rates (sensitivity) and false positive rates (1-specificity). This provides a robust measure of diagnostic accuracy, particularly in situations where the optimal classification threshold may vary. All statistical tests were two-tailed, and the significant p-value threshold was set at 0.05.

## Results

### Demographic, clinical, and cognitive features of the study groups

Summary statistics on demographics are shown in Table 1 and Supplementary Tables 1–2. No significant difference across study groups (HC, s-MCI, c-MCI, and AD) was found on age, educational level, and sex.

The baseline behavioral analysis (Table 1; Supplementary Tables 1 and 2) indicated that the s-MCI and the c-MCI groups displayed differences in general cognition (i.e., ADAS-11/13, MMSE) and memory (i.e., ADASQ4, RAVLT, LM-IR, and LM-DR). No further significant difference was observed between the MCI groups for neuropsychological and neuropsychiatric variables. Compared with the HC subjects, both the c-MCI subsets and the AD group exhibited significant impairments in general cognition, memory functions, language (i.e., animal fluency), and executive functions (i.e., TMT-B). Subjects of the AD group also had higher impairment in visuospatial abilities (i.e., CT-Drawing), naming (i.e., Multilingual Naming Test), and neuropsychiatric conditions (i.e., NPI and the GDS). The s-MCI group showed general cognitive performance comparable to the HC group, although their memory performance was significantly compromised. They were characterized by severe deficits across multiple cognitive domains compared to HC and MCI subjects.

We further evaluated longitudinal variations in neuropsychological features across the HC and MCI groups (Table 2 and Supplementary Tables 3). As reported in Table 2, the c-MCI group, compared to the s-MCI group, exhibited more significant variations in global cognition measures as indicated by the ADAS-11/13, MOCA, and FAQ. Additionally, there was a significant variation in visuo-spatial abilities (i.e., Clock Test-Drawing) and neuropsychiatric symptoms (i.e., NPI total). The c-MCI, but not the s-MCI group, showed significant changes in test scores of memories (i.e., LM-IR), language/executive functions (i.e., animal fluency and TMT-B), and neuropsychiatric features (i.e., NPI total).

**Table 2:** Demographic. neuropsychological, and clinical features of HC and MCI groups after follow-up

**Variable**	**HC**		**s-MCI**		**c-MCI**		**ANOVAs**		**Post-hoc comparisons**		
									**HC**		**s-MCI**
	**Mean**	**SD**	**Mean**	**SD**	**Mean**	**SD**	**F**	**p**	**s-MCI**	**c-MCI**	**c-MCI**
CDR-SB	0.10	0.27	0.32	1.37	2.71	1.96	59.89	**PP**	0.227	PP	PP
ADAS11	0.07	2.92	1.52	3.39	5.68	5.57	19.62	**PP**	0.428	PP	**0.004**
ADAS13	0.89	4.02	2.95	4.59	7.32	6.90	15.79	**PP**	0.355	PP	**0.022**
ADASQ4	0.80	3.48	0.52	3.40	1.63	3.56	1.52	0.224	0.944	0.195	0.624
MMSE	0.61	1.54	1.14	1.91	1.29	1.75	12.25	**PP**	0.315	**PP**	0.084
MoCA	0.69	2.37	0.19	2.40	3.06	2.94	8.99	**PP**	0.966	**PP**	**0.008**
FAQ	0.47	1.50	1.05	1.80	2.80	3.66	37.92	**PP**	0.875	**PP**	**PP**
RAVLT-IR	0.39	2.88	0.63	3.09	8.44	6.12	1.42	0.246	0.857	0.218	0.764
RAVLT-L	1.92	7.74	2.90	9.63	4.82	7.11	4.78	**0.010**	0.441	0.008	0.556
RAVLT-DR	-0.28	2.74	0.38	2.50	1.15	2.12	0.24	0.790	0.852	0.953	0.772
RAVLT-TOT	0.70	3.97	1.38	4.89	0.57	1.82	0.43	0.651	0.965	0.696	0.708
RAVLT-RN	0.70	2.49	0.76	3.21	1.06	3.83	0.42	0.658	0.968	0.700	0.716
LM-IR	-0.24	3.06	1.82	3.87	1.62	3.81	3.45	**0.035**	0.322	0.040	0.900
LM-DR	-0.01	2.82	2.41	3.70	1.47	4.63	3.17	**0.046**	0.198	0.080	1.000
CT-Drawing	-0.01	0.51	-0.05	1.21	0.71	1.27	7.84	**0.001**	0.858	0.001	**0.008**
CT-Copy	-0.10	0.62	-0.18	0.66	0.11	0.93	0.23	0.798	0.999	0.785	0.906
TMT-A	5.61	12.01	3.77	9.46	8.24	20.98	1.08	0.345	0.493	0.794	0.312
TMT-B	13.36	29.57	9.68	30.96	49.97	61.81	6.56	**0.002**	0.980	0.002	0.057
AF	0.58	5.29	1.41	3.33	3.60	3.30	3.77	**0.026**	0.855	0.019	0.322
MINT-CUE	-0.23	3.55	0.14	0.71	-0.49	2.05	0.37	0.692	0.806	0.902	0.667
MINT-Total	-0.16	5.18	-0.86	7.47	2.71	5.64	3.18	**0.045**	0.947	0.036	0.307
MINT-UNC	0.07	3.72	0.14	1.98	2.37	4.39	3.83	**0.025**	0.997	0.023	0.132
NPI-total	0.28	3.77	-0.55	5.35	3.91	8.38	7.72	**0.001**	0.915	0.001	**0.011**
GDS	0.76	1.89	0.36	1.43	0.59	2.20	0.15	0.862	0.869	0.947	0.972

Abbreviations: ADAS=Alzheimer’s Disease Assessment Scale (T1 items and 13 items versions); ADAS-Q4=ADAS delayed word recall subscale; AF=Animal Fluency; AVDEL-TOT=Rey Auditory Verbal Learning Test (Trials 1–6); CDR-RS=Clinical Dementia Rating Scale; CT-Copy=Clock Test- Copy score; CT-Drawing=Clock Test-Drawing total score; c-MCI=patients with MCI who convert to AD within 48-month follow-up; FAQ=Functional Activities Questionnaire; GDS=Geriatric Depression Scale; HC=healthy control stable after 48 months of follow-up; LM-IR=Logical Memory-Immediate Recall Total Number of Story Units Recalled; LM-DR=Logical Memory-Delayed Recall Total Number of Story Units Recalled; MINT-CUE=Multilingual Naming Test Total Correct - with Semantic Cue; MINT-Total=Multilingual Naming Test Total Correct (Uncued + Correct with Semantic cue); MINT-UNC =Multilingual Naming Test Total Uncued Correct; MMSE=Mini-Mental State Examination Total Score; MoCA=Montreal Cognitive Assessment; NPI=Neuropsychiatric Inventory Questionnaire; RAVLT-IR=Rey’s Auditory Verbal Learning Test, Immediate Recall (sum of 5 trials); RAVLT-L=Rey’s Auditory Verbal Learning Test, learning (trial 5 - trial 1); RAVLT-DR=Rey’s Auditory Verbal Learning Test, 30 minute Delayed Recall; RAVLT-RN=Rey’s Auditory Verbal Learning Test Delayed Recognition (RAVLT TOT Recognition Score - Total Intrusions); RAVLT-TOT= Rey’s Auditory Verbal Learning Test Total Recognition Score; s-MCI=MCI patients who did not convert to AD after 48-month follow-up; TMT=Trail Making Test (parts A and B).

### MRI volumetry of thalamic nuclei and whole thalamus

The main findings on MRI measures on thalamic nuclei are shown in Table 3. The results of the MANCOVA analysis revealed that, in comparison to HC subjects, both c-MCI individuals and AD patients exhibited widespread atrophy across the thalamic subfields. At the same time, the s-MCI group did not show significant differences (Supplementary Table 4). When comparing the s-MCI and c-MCI subjects, the c-MCI group showed significant atrophy in the nucleus reuniens and a trend toward significant atrophy in the AV and LD nuclei (Tables 3). The overall comparison of the entire thalamus did not yield significant differences, except when comparing AD subjects to HC subjects (Supplementary Tables 5 and 6).

**Table 3:** Thalamic nuclei volumetry for each study group

**Group**	**Nucleus**	**s-HC**		**s-MCI**		**c-MCI**		**AD**		**ANOVAs**		**s-MCI vs. c-MCI**
		**Mean**	**SD**	**Mean**	**SD**	**Mean**	**SD**	**Mean**	**SD**	**F**	**p**	
ANT	L-AV	8.63	1.62	8.56	1.20	7.35	1.44	7.69	2.06	6.888	**PP**	0.057
	R-AV	9.68	1.61	9.68	1.44	8.91	1.39	8.89	2.18	3.636	**0.014**	0.411
Dorsal	L-LD	1.80	0.61	1.69	0.57	1.23	0.64	1.23	0.75	13.564	**PP**	0.054
	R-LD	1.76	0.66	1.61	0.53	1.29	0.65	1.42	0.75	5.242	**0.002**	0.287
	L-LP	7.95	1.43	7.73	1.75	6.97	1.29	7.16	1.74	3.708	**0.012**	0.301
	R-LP	7.63	1.43	7.72	1.47	6.74	1.30	7.12	1.76	5.617	**0.001**	0.103
Ventral	L-VA	25.7	3.01	25.7	3.30	23.9	2.86	23.3	3.36	10.142	**PP**	0.158
	L-VAmc	1.93	0.22	1.91	0.28	1.79	0.21	1.80	0.27	5.571	**0.001**	0.272
	L-VLa	38.2	3.87	38.0	4.88	36.4	4.58	35.9	4.49	4.701	**0.003**	0.512
	L-VLp	50.2	4.93	49.7	6.49	48.1	6.34	48.1	6.02	2.460	0.063	NA
	L-VPL	55.1	5.84	54.6	8.19	54.1	7.91	54.2	7.50	0.346	0.792	NA
	L-VM	1.37	0.23	1.35	0.21	1.32	0.20	1.32	0.21	0.001	1.000	NA
	R-VA	25.6	2.57	26.0	3.44	24.2	3.03	23.4	3.33	10.645	**PP**	0.126
	R-VAmc	2.01	0.23	2.04	0.31	1.91	0.23	1.89	0.28	4.529	**0.004**	0.233
	R-VLa	39.1	3.81	39.2	5.72	37.6	5.13	37.1	4.78	3.531	**0.016**	0.570
	R-VLp	51.0	5.29	50.7	7.62	49.2	6.61	49.4	6.41	1.331	0.265	NA
	R-VPL	55.5	6.81	54.1	9.29	53.8	7.29	55.7	8.38	0.721	0.540	NA
	R-VM	1.41	0.27	1.35	0.30	1.35	0.21	1.38	0.25	0.001	1.000	NA
NS	L-CeM	4.11	0.73	4.06	0.77	3.59	0.67	3.56	0.94	8.386	**PP**	0.154
	L-CL	2.32	0.61	2.15	0.50	2.04	0.57	2.18	0.69	0.572	0.634	NA
	L-CM	15.2	1.49	15.5	2.29	15.0	2.03	15.0	1.91	1.852	0.138	NA
	L-Pc	0.22	0.03	0.22	0.04	0.02	0.04	0.02	0.03	0.001	1.000	NA
	L-Pf	3.53	0.40	3.57	0.53	3.47	0.52	3.41	0.48	1.384	0.248	NA
	R-CeM	4.28	0.74	4.40	0.76	3.90	0.77	3.73	0.98	7.975	**PP**	0.149
	R-CL	2.32	0.62	2.25	0.56	2.19	0.54	2.33	0.66	0.546	0.652	NA
	R-CM	15.5	1.69	15.3	2.48	15.1	1.91	15.3	2.08	0.277	0.842	NA
	R-Pc	0.25	0.04	0.24	0.49	0.24	0.04	0.23	0.042	0.001	1.000	0.999
	R-Pf	3.75	0.45	3.78	0.60	3.67	0.55	3.56	0.59	2.388	0.070	NA
Medial	L-Re	0.72	0.20	0.73	0.18	0.59	0.17	0.56	0.23	10,456	**PP**	0,058
	R-Re	0.76	0.22	0.81	0.21	0.60	0.21	0.57	0.24	15,245	**PP**	**0,007**
	L-MDm	4.65	6.46	4.46	8.97	4.25	8.49	4.18	7.31	6,628	**PP**	0,714
	R-MDm	4.64	6.86	4.40	9.14	4.23	7.74	4.14	7.42	7,457	**PP**	0,834
	L-MDl	1.73	2.51	1.72	3.09	1.57	2.80	1.50	2.97	11,299	**PP**	0,194
	R-MDl	1.74	2.58	1.70	3.04	1.54	3.08	1.48	3.13	12,707	**PP**	0,182
	L-Pt	0.45	0.07	0.45	0.06	0.44	0.07	0.45	0.07	0,000	1,000	NA
	R-Pt	0.47	0.06	0.45	0.07	0.45	0.06	0.46	0.07	0.001	1.000	NA
PUL	L-PuA	13.5	1.60	13.4	2.49	12.5	1.49	12.5	1.93	6.086	**0.001**	0.247
	L-PuI	14.8	2.15	15.0	3.23	14.2	1.78	13.8	2.42	3.919	**0.009**	0.555
	L-PuL	12.6	2.49	13.3	3.45	12.3	1.97	12.2	2.48	1.133	0.337	NA
	L-PuM	69.4	7.71	68.7	1.26	65.7	7.26	66.2	9.21	2.615	0.052	NA
	R-PuA	13.8	1.47	13.1	2.21	12.4	1.57	12.5	2.00	9.774	**PP**	0.462
	R-PuI	15.1	2.40	14.4	2.46	14.1	2.27	14.0	2.47	3.665	**0.013**	0.976
	R-PuL	13.0	2.71	13.1	2.42	12.2	2.22	12.1	2.29	2.611	0.052	NA
	R-PuM	71.2	7.44	67.2	1.05	65.7	7.32	67.0	9.47	5.091	0.002	0.921
MTH	L-LGN	15.1	2.47	1.51	3.22	1.39	2.45	1.34	2.17	8.815	PP	0.215
	L-MGN	7.25	1.13	7.39	1.20	7.05	1.10	6.98	1.16	1.282	0.281	NA
	L-SG	1.70	0.43	1.63	0.49	1.68	0.29	1.69	0.42	0.001	1.000	NA
	R-LGN	14.8	2.38	14.9	3.62	13.8	2.00	13.8	2.24	3.862	0.010	0.326
	R-MGN	8.06	1.28	8.14	1.33	7.86	1.28	8.16	1.53	0.432	0.730	NA
	R-SG	1.61	0.37	1.55	0.36	1.54	0.32	1.63	0.42	0.001	1.000	NA

Values x 10-4 are expressed as the mean &plusmn; standard deviation (SD). MANOVA’s outputs: F= 2.061, PP. Bold values are statistically significant comparisons in ANOVAs and post-hoc comparisons. Abbreviations: AD=Alzheimer’s disease; ANT=anterior group; AV=anterior; CeM=central medial; CL=central lateral; c-MCI=patients with MCI who convert to AD within 48-month follow-up; CM=centromedian; HC=healthy control stable after 48 months of follow-up; LD=laterodorsal; LP=lateroposterior (LP) nuclei; LGN=lateral geniculate nucleus; MGN=media geniculate nucleus; MDm=magnocellular medial mediodorsal; MDl= parvocellular lateral mediodorsal; MTH=metathalamus; Re=reuniens; NA=Not Applicable; NS=non-specific nuclei; Pc=paracentral; Pf=parafascicular; Pt=paratenial; PuA=anterior pulvinar; PuI=inferior pulvinar; PuL=lateral pulvinar; PuM=medial pulvinar; s-MCI=MCI patients who did not convert to AD after 48-month follow-up; SG=limitans; VA=ventral-anterior; VLa=ventrolateral anterior; VLp=posterior, VPL=ventral-postero-lateral; VM=ventromedial nuclei.

### Discriminant function and ROC analyses

The discriminant function analysis showed that the right nucleus reuniens was the most significant predictor for distinguishing diagnostic groups, maximizing the separation between c-MCI and s-MCI subjects (Supplementary Table 7). The area under the curve (AUC) for discriminating c-MCI from s-MCI based on the volume of the nucleus reuniens was 0.76, with a sensitivity of 0.73 and specificity of 0.69. As expected, the AUC for the nucleus reuniens was higher than the AUCs calculated for any other thalamic nucleus or the whole thalamus (as shown in Supplementary Table 8).

## Discussion

The primary aim of the present study was to detect morphometric alterations in specific thalamic nuclei in patients encompassing the AD spectrum. While AD individuals exhibited generalized atrophy of the whole thalamus, our intergroup analysis revealed a distinct morphometric pattern that characterizes the transition from MCI to AD. The analysis points to the crucial involvement of the nucleus reuniens. Additionally, a trend toward a significant difference between c-MCI and s-MCI was observed for the limbic thalamus, including the AV and LD nuclei. Consistently, the discriminant function analysis confirmed the atrophy of the nucleus reuniens as the most influential predictor for distinguishing between the c-MCI and s-MCI groups.

**Figure 2:**
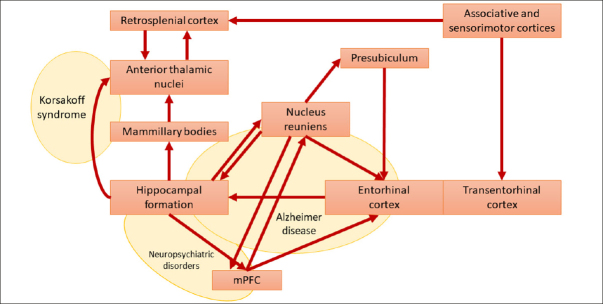
Papez-reuniens circuit in red [as per Braak and Braak nomenclature] and some possible lesion-derived clinical syndromes in yellow

Modified from (19). Abbreviation: mPFC, medial prefrontal cortex

A broad alteration of the Papez circuit has been reported in various conditions, including AD, Amyotrophic lateral sclerosis (ALS), Korsakoff syndrome, depression, Parkinson’s disease, epilepsy, and schizophrenia (19, 39–44). From a physiological standpoint, the nucleus reuniens represents a central hub within the Papez circuit (9, 45, 46), serving as the principal source of thalamic input to the hippocampus (47). Given the absence of direct bidirectional anatomical connectivity between these two structures, this nucleus functions as a crucial glutamatergic relay, facilitating the interaction between the medial prefrontal cortex (mPFC) and the hippocampus. By receiving inputs from the mPFC, the nucleus reuniens acts as a filter, contributing to memory consolidation and spatial navigation through excitatory projections to the hippocampus and presubiculum (21) (Figure 2). This mechanism remains active during slow-wave sleep (48, 49). Thirty years ago, Braak & Braak (19, 20, 39) first recognized the importance of the nucleus reuniens in the pathophysiology of AD. Their research demonstrated that the nucleus reuniens contains virtually no amyloid plaques but shows an abundance of neurofibrillary tangles (19). This phenomenon is associated with deficits in memory consolidation and spatial navigation that typically affect subjects at the early AD stages. More recently, atrophy of the nucleus reuniens has been described in ALS and AD (14, 40). Likewise, accumulation of neurofibrillary tangles and associated neurodegeneration in the nucleus reuniens has been observed in Guam’s ALS/Parkinsonism-Dementia Complex (50). Interestingly, a recent study demonstrated how deep brain stimulation of the nucleus reuniens promotes neuronal and cognitive resilience in an AD mouse model (51).

**Figure 3:**
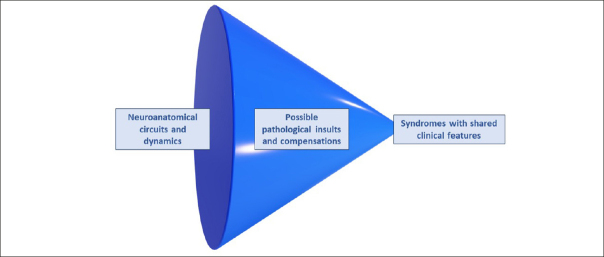
Clinicopathological bottleneck showing how different diseases share converging common manifestations I-1

While the syndromes mentioned above differ in their clinical presentations, they share progressive decline in mnemonic and cognitive functions. Therefore, the Papez circuit can be affected in various ways, but the resulting clinical symptoms are more homogeneous in their expression. This clinicopathological bottleneck (Figure 3) may help elucidate converging clinical manifestations of different neurological diseases.

In simpler terms, while the connectivity of cortical and subcortical structures (and their potential deficits) is multidimensional, the clinical expression exhibits lower dimensionality (52). Considering the anatomopathological staging proposed by Braak and Braak, neuropathological changes in the nucleus reuniens emerge in stages 3–4, overlapping with marked deterioration of symptoms (20). Intriguingly, Braak & Braak’s studies revealed that the pace of neurofibrillary tangles accumulation in the nucleus reuniens mirrors what found in mammillary bodies, subiculum, and entorhinal cortex (20). Therefore, the degeneration of the nucleus reuniens and its connections could be viewed as one of the critical tipping points for AD progression, when the circuit–s topology and dynamics can no longer compensate for ongoing degeneration (53–55).

Another interesting result of the present study is the tendency for significant atrophy of the AV nuclei in cMCI compared to sMCI. The AV nuclei and nucleus reuniens share connections with cortical and subcortical structures but, despite their apparent functional similarities, do exhibit specific differences. According to the model proposed by Mathiasen and colleagues (56), the AV contributes to developing a spatial navigation system through its connections with the hippocampus. This function is also crucial for navigation in the mental world (e.g., episodic memory, (57)). Compared to the AV, the output of the nucleus reuniens to the hippocampus is less stable, suggesting a role in the flexible updating of the map constructed through AV. Based on this, the spatial gradient of morphometric alterations observed in the thalamus might be associated with the initial cognitive deterioration observed in MCI. While the AV nucleus is only partially affected, making it still possible to use spatial/cognitive maps, the evident atrophy of the nucleus reuniens would specifically impair the flexibility in using these maps. While we did not observe a significant correlation between MRI and neuropsychological outcomes, the present speculation might be tested using more sensitive and longitudinal cognitive evaluations that distinguish the capacity to form a map from the ability to use it flexibly.

The present study has several strengths. Firstly, fully automated algorithms for anatomical parcellation and measurement of pathological changes in thalamic nuclei, implemented in Freesurfer v.7.3, ensure consistent and dependable measurements. This is particularly advantageous when dealing with large sample sizes and regular clinical settings (58–60). Secondly, the substantial sample drawn from the multimodal ADNI-3 database allowed stringent screening criteria and a 4-year clinical follow-up, ensuring diagnostic accuracy within the HC and s-MCI groups. Third, the integration of probabilistic techniques and a priori information from ex-vivo MRI and histology to analyze MRI raw data enabled a precise evaluation of relatively small thalamic nuclei, such as the nucleus reuniens, which are difficult to isolate using standard imaging data analysis techniques. At the same time, several limitations of the study should be acknowledged. First, the cross-sectional design fails to capture longitudinal shifts in brain atrophy, which could provide invaluable insights into forecasting the progression to more advanced disease stages. The inclusion of longitudinal MRI analyses would allow more comprehensive understanding of the timing, spatial distribution, and advancement of hippocampal and amygdala involvement in AD. Second, a more precise segmentation could be achieved using scanners with higher fields (e.g., 7T). However, no patient database with this critical feature is currently available. Third, the sample size used in the present study was relatively small. Our results must be replicated in a greater sample when larger databases are made available. Notably, we deliberately chose to include only subjects with complete information at baseline and 48 months. We believe that our stringent inclusion criteria enhance our database’s quality and our findings’ accuracy. Moreover, we acknowledge that the small sample size of s-MCI might influence the negative results. Nevertheless, the presence of a significant (and robust) effect between s-MCI and c-MCI, which is the key finding of our study, suggests that the numerosity of the s-MCI, affected by our stringent inclusion criteria, does not hinder the detection of structural differences, when present.

In conclusion, understanding the intricate dynamics of cortico-subcortical dysfunction upon the clinical transition to AD provides valuable information for optimizing clinical approaches and identifying potential MCI subjects at risk of AD progression.

### Electronic supplementary material

Supplementary material, approximately 51.2 KB.

## Notes

*Disclosure statement:* Valentina Tomassini has received honoraria, travel grants, and research grant support from FISM, the Italian Ministry of Health, Alexion, Roche, Merck, Biogen, Novartis, Viatris, Bristol Myers Squibb, Almirall, Horizon, Lundbeck, Sanofi, Janssen.

## Notes

*Ethical standards:* ADNI obtained all IRB approvals and met all ethical standards in the collection of data. The following are the ethics committees and IRB boards that provided approval.
