# Supplementary material for: “Back to Braak”: Role of Nucleus Reuniens and Subcortical Pathways in Alzheimer's Disease Progression
Source: J Prev Alzheimers Dis. 2024 Feb 21;11(4):1030–40. doi: 10.14283/jpad.2024.42 (PMC11266379; doi:10.14283/jpad.2024.42)
Supplement: Supplementary file 1 — Supplementary material, approximately 51.2 KB. [file mmc1.docx]

**Supplementary Table 1.** Tukey's post-hoc comparisons between the MCI subsets with HC and AD groups.

| Variable | HC vs. | | | s-MCI | c-MCI |
| --- | --- | --- | --- | --- | --- |
|  | s-MCI | c-MCI | AD | AD | AD |
| CDR-RS | **.001** | **PP** | **PP** | **PP** | **PP** |
| ADAS-11 | **.017** | **PP** | **PP** | **PP** | **PP** |
| ADAS-13 | **.003** | **PP** | **PP** | **PP** | **PP** |
| ADAS-Q4 | .580 | **PP** | **PP** | **PP** | **PP** |
| MMSE | .232 | **PP** | **PP** | **PP** | **PP** |
| MoCA | **PP** | **PP** | **PP** | **PP** | **PP** |
| FAQ | **.002** | **PP** | **PP** | **PP** | .056 |
| RAVLT-DR | .802 | **PP** | **PP** | **PP** | .166 |
| RAVLT-IR | **.001** | **PP** | **PP** | **PP** | .281 |
| RAVLT-L | .305 | **PP** | **PP** | **PP** | **.002** |
| RAVLT-RN | .087 | **PP** | **PP** | **PP** | **.002** |
| RAVLT-TOT | **.006** | **PP** | **PP** | **PP** | **PP** |
| LM-IR | **.006** | **PP** | **PP** | **PP** | **.001** |
| LM-DR | **.001** | **PP** | **PP** | **PP** | **PP** |
| CT-Drawing | **.038** | **.032** | **PP** | .135 | **.019** |
| TMT-A | .896 | .895 | **.001** | .247 | .101 |
| TMT-B | .929 | **.008** | **PP** | PP | .065 |
| AF | .276 | **PP** | **PP** | PP | **PP** |
| MINT-UNC | **.002** | **PP** | **PP** | PP | **.016** |
| MINT-Total | .951 | .379 | **.049** | .654 | .981 |
| NPI-Total | .259 | .270 | **PP** | .137 | **.023** |
| GDS | .368 | **.007** | **PP** | **.005** | **.046** |

Bold values are statistically significant comparisons.

*Abbreviations*: ADAS=Alzheimer’s Disease Assessment Scale (11 items and 13 items versions); ADAS-Q4=ADAS delayed word recall subscale; AF=Animal Fluency; ANOVA=Analysis Of Variance; CDR-RS=Clinical Dementia Rating Scale; CT- Drawing=Clock Test- Drawing total score; c-MCI=patients with MCI who convert to AD within 48-month follow-up; FAQ=Functional Activities Questionnaire; GDS=Geriatric Depression Scale; HC=healthy control stable after 48 months of follow-up; LM-IR=Logical Memory-Immediate Recall Total Number of Story Units Recalled; LM-DR=Logical Memory-Delayed Recall Total Number of Story Units Recalled; MINT-Total=Multilingual Naming Test Total Correct (Uncued + Correct with Semantic cue); MINT-Uncued=Multilingual Naming Test Total Uncued Correct; MoCA=Montreal Cognitive Assessment; NPI=Neuropsychiatric Inventory Questionnaire; RAVLT-IR=Rey’s Auditory Verbal Learning Test. Immediate Recall (sum of 5 trials); RAVLT-L=Rey's Auditory Verbal Learning Test. learning (trial 5 - trial 1); RAVLT-DR=Rey’s Auditory Verbal Learning Test. 30 minute Delayed Recall; RAVLT-RN=Rey’s Auditory Verbal Learning Test Delayed Recognition(RAVLT TOT Recognition Score – Total Intrusions); RAVLT-TOT=Rey’s Auditory Verbal Learning Test Total Recognition Score; s-MCI=patients with MCI who remain stable within 48-month follow-up.

**Supplementary Table 2.** Data availability for demographic and neuropsychological information at baseline.

| Variable | HC | s-MCI | c-MCI | AD | Total subjects |
| --- | --- | --- | --- | --- | --- |
| Age | 84 | 22 | 36 | 102 | 242 |
| Educational level | 84 | 22 | 36 | 102 | 242 |
| CDR-RS | 84 | 22 | 34 | 102 | 241 |
| ADAS-11 | 84 | 22 | 36 | 99 | 239 |
| ADAS-13 | 84 | 22 | 36 | 99 | 239 |
| ADAS-Q4 | 84 | 22 | 36 | 102 | 242 |
| MMSE | 84 | 21 | 35 | 102 | 241 |
| MoCA | 81 | 22 | 35 | 96 | 232 |
| FAQ | 84 | 22 | 35 | 100 | 239 |
| RAVLT-DR | 84 | 22 | 36 | 99 | 239 |
| RAVLT-IR | 84 | 22 | 36 | 99 | 239 |
| RAVLT-L | 84 | 22 | 36 | 99 | 239 |
| RAVLT-RN | 84 | 22 | 36 | 99 | 239 |
| RAVLT-TOT | 84 | 22 | 36 | 99 | 239 |
| LM-IR | 84 | 22 | 36 | 101 | 241 |
| LM-DR | 84 | 22 | 36 | 100 | 240 |
| CT-Drawing | 84 | 22 | 36 | 100 | 240 |
| CT-Copy | 84 | 22 | 36 | 100 | 240 |
| TMT-A | 84 | 22 | 36 | 98 | 238 |
| TMT-B | 84 | 22 | 36 | 88 | 228 |
| AF | 84 | 22 | 36 | 100 | 240 |
| MINT-SEM | 83 | 22 | 36 | 98 | 237 |
| MINT-UNC | 83 | 22 | 36 | 98 | 237 |
| MINT-Total | 83 | 22 | 36 | 98 | 237 |
| NPI-Total | 84 | 22 | 36 | 102 | 242 |
| GDS | 84 | 22 | 35 | 99 | 238 |

*Abbreviations*: AD=Alzheimer’s disease; ADAS=Alzheimer’s Disease Assessment Scale (11 items and 13 items versions); ADAS-Q4=ADAS delayed word recall subscale; AF=Animal Fluency; AVDEL-TOT=Rey Auditory Verbal Learning Test (Trials 1-6); CDR-RS=Clinical Dementia Rating Scale; CT-Copy=Clock Test- Copy score; CT- Drawing=Clock Test- Drawing total score; c-MCI=patients with MCI who convert to AD within 48-month follow-up; FAQ=Functional Activities Questionnaire; GDS=Geriatric Depression Scale; HC=healthy control stable after 48 months of follow-up; LM-IR=Logical Memory-Immediate Recall Total Number of Story Units Recalled; LM-DR=Logical Memory-Delayed Recall Total Number of Story Units Recalled; MINT-Semantic cue=Multilingual Naming Test Total Correct - with Semantic Cue; MINT-Total=Multilingual Naming Test Total Correct (Uncued + Correct with Semantic cue); MINT-UNC =Multilingual Naming Test Total Uncued Correct; MMSE=Mini-Mental State Examination Total Score; MoCA=Montreal Cognitive Assessment; NPI=Neuropsychiatric Inventory Questionnaire; RAVLT-IR=Rey’s Auditory Verbal Learning Test. Immediate Recall (sum of 5 trials); RAVLT-L=Rey's Auditory Verbal Learning Test. learning (trial 5 - trial 1); RAVLT-DR=Rey’s Auditory Verbal Learning Test 30 minute Delayed Recall; RAVLT-RN=Rey’s Auditory Verbal Learning Test Delayed Recognition (RAVLT TOT Recognition Score – Total Intrusions); RAVLT-TOT=Rey’s Auditory Verbal Learning Test Total Recognition Score; s-MCI=MCI patients who did not convert to AD after 48-month follow-up; TMT=Trail Making Test (parts A and B).

**Supplementary Table 3.** Data availability for neuropsychological information after follow-up.

| Variable | HC | s-MCI | c-MCI | Total |
| --- | --- | --- | --- | --- |
| CDR-SB | 81 | 19 | 34 | 134 |
| ADAS-11 | 82 | 21 | 34 | 137 |
| ADAS-13 | 82 | 21 | 33 | 136 |
| ADAS-Q4 | 83 | 21 | 35 | 139 |
| MMSE | 83 | 22 | 34 | 139 |
| MoCA | 80 | 21 | 33 | 134 |
| FAQ | 81 | 21 | 35 | 137 |
| RAVLT-DR | 79 | 19 | 34 | 132 |
| RAVLT-IR | 83 | 21 | 34 | 138 |
| RAVLT-L | 83 | 21 | 34 | 138 |
| RAVLT-RN | 83 | 21 | 35 | 139 |
| RAVLT-TOT | 83 | 21 | 35 | 139 |
| LM-IR | 83 | 22 | 34 | 139 |
| LM-DR | 83 | 22 | 34 | 139 |
| CT-Drawing | 83 | 22 | 35 | 140 |
| CT-Copy | 83 | 22 | 35 | 140 |
| TMT-A | 83 | 22 | 34 | 139 |
| TMT-B | 83 | 22 | 33 | 138 |
| AF | 83 | 22 | 35 | 140 |
| MINT-SEM | 81 | 22 | 35 | 138 |
| MINT-UNC | 82 | 22 | 35 | 139 |
| MINT-TOTAL | 82 | 22 | 35 | 139 |
| NPI-TOTAL | 83 | 22 | 35 | 140 |
| GDS | 82 | 22 | 34 | 138 |

*Abbreviations*: ADAS=Alzheimer’s Disease Assessment Scale (11 items and 13 items versions); ADAS-Q4=ADAS delayed word recall subscale; AF=Animal Fluency; AVDEL-TOT=Rey Auditory Verbal Learning Test (Trials 1-6); CDR-RS=Clinical Dementia Rating Scale; CT-Copy=Clock Test- Copy score; CT- Drawing=Clock Test- Drawing total score; c-MCI=patients with MCI who convert to AD within 48-month follow-up; FAQ=Functional Activities Questionnaire; GDS=Geriatric Depression Scale; HC=healthy control stable after 48 months of follow-up; LM-IR=Logical Memory-Immediate Recall Total Number of Story Units Recalled; LM-DR=Logical Memory-Delayed Recall Total Number of Story Units Recalled; MINT-Semantic cue=Multilingual Naming Test Total Correct - with Semantic Cue; MINT-Total=Multilingual Naming Test Total Correct (Uncued + Correct with Semantic cue); MINT-UNC =Multilingual Naming Test Total Uncued Correct; MMSE=Mini-Mental State Examination Total Score; MoCA=Montreal Cognitive Assessment; NPI=Neuropsychiatric Inventory Questionnaire; RAVLT-IR=Rey’s Auditory Verbal Learning Test. Immediate Recall (sum of 5 trials); RAVLT-L=Rey's Auditory Verbal Learning Test. learning (trial 5 - trial 1); RAVLT-DR=Rey’s Auditory Verbal Learning Test. 30 minute Delayed Recall; RAVLT-RN=Rey’s Auditory Verbal Learning Test Delayed Recognition (RAVLT TOT Recognition Score – Total Intrusions); RAVLT-TOT=Rey’s Auditory Verbal Learning Test Total Recognition Score; s-MCI=MCI patients who did not convert to AD after 48-month follow-up; TMT=Trail Making Test (parts A and B).

**Supplementary Tables 4.** Tukey's post-hoc comparisons between the MCI subsets with HC and AD groups for thalamic nuclei.

| Group | Nucleus | HC vs. | | | AD vs. | |
| --- | --- | --- | --- | --- | --- | --- |
|  |  | s-MCI | c-MCI | AD | s-MCI | c-MCI |
| Anterior | L-AV | 0.999 | **0.002** | **0.002** | 0.157 | 0.753 |
|  | R-AV | 1.000 | 0.155 | **0.020** | 0.265 | 1.000 |
| Dorsal | L-LD | 0.901 | **PP** | **PP** | **0.020** | 1.000 |
|  | L-LP | 0.939 | **0.011** | **0.005** | 0.424 | 0.922 |
|  | R-LD | 0.813 | **0.004** | **0.005** | 0.618 | 0.769 |
|  | R-LP | 0.996 | **0.025** | 0.126 | 0.377 | 0.598 |
| Ventral | L-VA | 1.000 | **0.032** | **PP** | **0.007** | 0.735 |
|  | L-VAmc | 0.990 | **0.026** | **0.002** | 0.205 | 0.999 |
|  | L-VLa | 0.998 | 0.159 | **0.003** | 0.188 | 0.958 |
|  | R-VA | 0.966 | 0.075 | **PP** | **0.002** | 0.511 |
|  | R-VAmc | 0.934 | 0.247 | **0.011** | 0.054 | 0.968 |
|  | R-VLa | 1.000 | 0.349 | **0.017** | 0.212 | 0.944 |
| Non-specific | L-CeM | 0.996 | **0.010** | **PP** | **0.046** | 0.996 |
|  | R-CeM | 0.939 | 0.130 | **PP** | **0.006** | 0.728 |
|  | R-Pc | 0.590 | 0.316 | **0.003** | 0.796 | 0.805 |
| Medial | L-MVRe | 0.991 | **0.012** | **PP** | **0.004** | 0.925 |
|  | L-MDm | 0.726 | **0.038** | **PP** | 0.361 | 0.959 |
|  | L-MDl | 0.999 | **0.023** | **PP** | **0.007** | 0.634 |
|  | R-MVRe | 0.874 | **0.003** | **PP** | **PP** | 0.837 |
|  | R-MDm | 0.519 | **0.029** | **PP** | 0.444 | 0.922 |
|  | R-MDl | 0.964 | **0.006** | **PP** | **0.009** | 0.738 |
| Pulvinar | L-PuA | 0.999 | **0.039** | **0.001** | 0.106 | 0.998 |
|  | L-PuI | 0.995 | 0.442 | **0.011** | 0.128 | 0.841 |
|  | R-PuA | 0.367 | **0.001** | **PP** | 0.459 | 0.994 |
|  | R-PuI | 0.587 | 0.163 | **0.008** | 0.889 | 0.991 |
|  | R-PuM | 0.217 | **0.009** | **0.006** | 1.000 | 0.864 |
| Metathalamus | L-LGN | 1.000 | **0.048** | **PP** | **0.016** | 0.789 |
|  | R-LGN | 0.999 | 0.147 | **0.018** | 0.191 | 1.000 |

Bold values are statistically significant comparisons in post-hoc comparisons.

*Abbreviations:* AD=Alzheimer’s disease; ANT=anterior group; AV=anterior; CeM=central medial; CL=central lateral; c-MCI=patients with MCI who convert to AD within 48-month follow-up; CM=centromedian; HC=healthy control stable after 48 months of follow-up; L=Left; LD=laterodorsal; LP=lateroposterior (LP) nuclei; LGN=lateral geniculate nucleus; MGN=media geniculate nucleus; MDm=magnocellular medial mediodorsal; MDl= parvocellular lateral mediodorsal; MTH=metathalamus; R=Right; Re=reuniens; NA=Not Applicable; NS=non-specific nuclei; Pc=paracentral; Pf=parafascicular; Pt=paratenial; PuA=anterior pulvinar; PuI=inferior pulvinar; PuL=lateral pulvinar; PuM=medial pulvinar; s-MCI=MCI patients who did not convert to AD after 48-month follow-up; SG=limitans; VA=ventral-anterior; VLa=ventrolateral anterior; VLp=posterior, VPL=ventral-postero-lateral; VM=ventromedial nuclei.

**Supplementary Tables 5.** Statistics on left and right whole thalamus

| Variable | HC | | s-MCI | | c-MCI | | AD | |
| --- | --- | --- | --- | --- | --- | --- | --- | --- |
|  | Mean | SD | Mean | SD | Mean | SD | Mean | SD |
| eTIV (mm^3^) | 1494125 | 161017 | 1489721 | 264527 | 1538897 | 211560 | 1519689 | 204803 |
| LWT/eTIV | 0.0051 | 0.0007 | 0.0051 | 0.0007 | 0.0049 | 0.0008 | 0.0046 | 0.0010 |
| RWT/eTIV | 0.0050 | 0.0007 | 0.00413 | 0.0007 | 0.0048 | 0.0007 | 0.0045 | 0.0010 |

*Abbreviations:* AD=Alzheimer’s disease; c-MCI=patients with MCI who convert to AD within 48-month follow-up; eTIV=estimated Total Intracranial Volume; HC=healthy control stable after 48 months of follow-up; LWT=left whole thalamus; RWT=right whole thalamus; s-MCI=MCI patients who did not convert to AD after 48-month follow-up.

**Supplementary Table 6.** Between-group comparison for left and right whole thalamus.

| Variable | HC vs. | | | s-MCI vs. | | c-MCI |
| --- | --- | --- | --- | --- | --- | --- |
|  | s-MCI | c-MCI | AD | c-MCI | AD | AD |
| LWT/eTIV | 0.997 | 0.563 | **PP** | 0.864 | 0.092 | 0.312 |
| RWT/eTIV | 1.000 | 0.605 | **0.001** | 0.764 | 0.079 | 0.419 |

MANOVA F= 3.962, p=0.001.

*Abbreviations:* AD=Alzheimer’s disease; c-MCI=patients with MCI who convert to AD within 48-month follow-up; eTIV=estimated Total Intracranial Volume; HC=healthy control stable after 48 months of follow-up; LWT=left whole thalamus; RWT=right whole thalamus; s-MCI=MCI patients who did not convert to AD after 48-month follow-up.

**Supplementary Table 7.** ROC analysis.

|  | AUC | SE | p | IC min | ICmax |
| --- | --- | --- | --- | --- | --- |
| **R-MVRe** | **0.759** | **0.066** | **0.001** | **0.63** | **0.888** |
| **L-AV** | **0.744** | **0.066** | **0.002** | **0.614** | **0.873** |
| **L-LD** | **0.734** | **0.069** | **0.003** | **0.6** | **0.869** |
| **L-VA** | **0.726** | **0.07** | **0.004** | **0.589** | **0.863** |
| **L-MVRe** | **0.724** | **0.071** | **0.004** | **0.584** | **0.864** |
| **R-VA** | **0.716** | **0.071** | **0.006** | **0.577** | **0.854** |
| **L-CeM** | **0.7** | **0.072** | **0.011** | **0.56** | **0.84** |
| **L-VAmc** | **0.691** | **0.073** | **0.016** | **0.547** | **0.834** |
| **R-CeM** | **0.686** | **0.074** | **0.018** | **0.54** | **0.832** |
| **R-LP** | **0.686** | **0.073** | **0.018** | **0.542** | **0.83** |
| **L-WT** | **0.672** | **0.074** | **0.029** | **0.526** | **0.817** |
| **R-LD** | **0.669** | **0.071** | **0.032** | **0.529** | **0.809** |
| **R-VAmc** | **0.666** | **0.077** | **0.035** | **0.514** | **0.818** |
| **R-AV** | **0.662** | **0.076** | **0.04** | **0.513** | **0.811** |
| L-PuA | 0.652 | 0.082 | 0.053 | 0.49 | 0.814 |
| L-MDl | 0.65 | 0.075 | 0.057 | 0.504 | 0.797 |
| L-VLa | 0.648 | 0.073 | 0.061 | 0.504 | 0.792 |
| R-MDl | 0.647 | 0.075 | 0.062 | 0.501 | 0.793 |
| L-VLp | 0.646 | 0.074 | 0.064 | 0.5 | 0.792 |
| L-LP | 0.641 | 0.079 | 0.074 | 0.486 | 0.795 |
| L-LGN | 0.634 | 0.078 | 0.088 | 0.483 | 0.786 |
| R-VLa | 0.634 | 0.077 | 0.089 | 0.483 | 0.785 |
| R-WT | 0.632 | 0.079 | 0.094 | 0.478 | 0.786 |
| R-PuL | 0.627 | 0.075 | 0.107 | 0.479 | 0.775 |
| R-Pf | 0.6 | 0.079 | 0.206 | 0.445 | 0.755 |
| R-VLp | 0.597 | 0.078 | 0.217 | 0.444 | 0.751 |
| L-PuI | 0.595 | 0.08 | 0.229 | 0.439 | 0.751 |
| L-CM | 0.593 | 0.077 | 0.239 | 0.443 | 0.743 |
| L-Pc | 0.593 | 0.076 | 0.239 | 0.443 | 0.743 |
| R-PuA | 0.593 | 0.08 | 0.236 | 0.436 | 0.751 |
| R-LGN | 0.591 | 0.083 | 0.249 | 0.427 | 0.755 |
| L-MGN | 0.591 | 0.077 | 0.249 | 0.44 | 0.742 |
| R-MGN | 0.59 | 0.078 | 0.252 | 0.438 | 0.742 |
| L-PuM | 0.588 | 0.081 | 0.265 | 0.429 | 0.746 |
| L-PuL | 0.585 | 0.082 | 0.279 | 0.425 | 0.745 |
| L-MDm | 0.569 | 0.079 | 0.382 | 0.415 | 0.723 |
| L-Pf | 0.566 | 0.077 | 0.405 | 0.415 | 0.716 |
| R-PuM | 0.563 | 0.081 | 0.428 | 0.403 | 0.722 |
| R-PuI | 0.556 | 0.079 | 0.481 | 0.402 | 0.709 |
| L-Pt | 0.554 | 0.077 | 0.496 | 0.402 | 0.705 |
| L-VPL | 0.553 | 0.077 | 0.501 | 0.402 | 0.704 |
| R-CM | 0.553 | 0.08 | 0.501 | 0.397 | 0.709 |
| L-VM | 0.552 | 0.076 | 0.506 | 0.403 | 0.702 |
| R-MDm | 0.552 | 0.081 | 0.506 | 0.394 | 0.71 |
| L-CL | 0.545 | 0.08 | 0.564 | 0.389 | 0.702 |
| R-VPL | 0.526 | 0.082 | 0.743 | 0.365 | 0.687 |
| R-CL | 0.525 | 0.08 | 0.749 | 0.369 | 0.682 |
| R-Pt | 0.525 | 0.079 | 0.749 | 0.37 | 0.681 |
| R-SG | 0.511 | 0.081 | 0.885 | 0.353 | 0.67 |
| R-Pc | 0.498 | 0.08 | 0.981 | 0.341 | 0.655 |
| R-VM | 0.494 | 0.081 | 0.943 | 0.335 | 0.654 |
| L-SG | 0.441 | 0.083 | 0.451 | 0.277 | 0.604 |

*Abbreviations:* AD=Alzheimer’s disease; ANT=anterior group; AV=anterior; CeM=central medial; CL=central lateral; c-MCI=patients with MCI who convert to AD within 48-month follow-up; CM=centromedian; HC=healthy control stable after 48 months of follow-up; L=left; LD=laterodorsal; LP=lateroposterior (LP) nuclei; LGN=lateral geniculate nucleus; MGN=media geniculate nucleus; MDm=magnocellular medial mediodorsal; MDl= parvocellular lateral mediodorsal; MTH=metathalamus; R=Right; Re=reuniens; NA=Not Applicable; NS=non-specific nuclei; Pc=paracentral; Pf=parafascicular; Pt=paratenial; PuA=anterior pulvinar; PuI=inferior pulvinar; PuL=lateral pulvinar; PuM=medial pulvinar; s-MCI=MCI patients who did not convert to AD after 48-month follow-up; SG=limitans; VA=ventral-anterior; VLa=ventrolateral anterior; VLp=posterior, VPL=ventral-postero-lateral; VM=ventromedial nuclei; WT=Whole Thalamus.
